# Effects of different fertilizer formulas on the growth of loquat rootstocks and stem lignification

**DOI:** 10.1038/s41598-019-57270-5

**Published:** 2020-01-23

**Authors:** Fangjie Xu, Changbin Chu, Zhihong Xu

**Affiliations:** 1 Forestry and Pomology Research Institute/Shanghai Key Lab of Protected Horticultural Technology, Shanghai Academy of Agricultural Sciences, No. 1000 Jinqi Road, Fengxian District, Shanghai, 201403 P.R. China; 2 Eco-Environmental Protection Institute, Shanghai Academy of Agricultural Sciences, No. 1000 Jinqi Road, Fengxian District, Shanghai, 201403 P.R. China; 3 College of Agricultural & Food Science, Zhejiang A & F University, No. 88 North Huancheng Road, Li’an District, Zhejiang, 311300 P.R. China

**Keywords:** Field trials, Plant physiology

## Abstract

Grafting is a common method of variety propagation in loquat breeding, the slow growth of rootstocks is a main factor limiting the expansion of this technique. This study aimed to evaluate the effects of seven different fertilizer formulas on the growth of loquat rootstock seedlings, five water-soluble fertilizer formulas, as well as organic fertilizer and controlled-release fertilizer were evaluated. An unfertilized control (CK) was also performed. Growth indicators including plant height, stem thickening and lignification, leaf area, root development, dry matter accumulation, spatial distribution of nutrient elements, and cross-sectional anatomy of stem were measured. The results showed that the addition of microelements in fertilizer could significantly delay the lignification process of the cambium, which exhibiting the greatest improvement in stem thickening. Phosphorus nutrition could significantly promote the occurrence of fibrous roots, while excessive phosphorus supply might disturb the absorption and utilization of nitrogen of roots, intensify the lignification process of the main stem, and then affect the growth of the aboveground part. The findings of this research could provide a theoretical basis for identifying an optimum fertilization formula and technique for promoting the rapid growth and accelerating the lignification process at different stages of loquat rootstock seedling growth.

## Introduction

Loquat (*Eriobotrya japonica* (Thunb.) Lindl., Maloideae, Rosaceae) is indigenous to China and represents one of the most important fruit species in subtropical areas of China^1^. Traditionally, there are several problems existed that limiting the propagation of loquat varieties, one is that seed reproduction takes too long juvenescent phase and seedling progeny cannot maintain variety characteristics due to genetic variation^2^, another problem is that explants are prone to browning in loquat asexual propagation system due to the higher content of phenols as compared with other Rosaceae fruit trees, therefore, callus proliferation needs strict selection of explants and proper medium. For example, Zhang^3^ reported that in tissue culture of loquat, leaf explants collected 5 days after bud sprouting were prone to severe browning and the highest mortality rate, whereas 10-day-old leaf explants showed minor browning symptoms and rapidly induced large quantity of granular light-green tight, high quality calluses, 15-day-old leaf explants also showed minor browning symptoms, but the generated calluses were yellow and loose, with lower proliferation efficiency. What is more, different varieties and ploidy materials of loquat have different requirements on medium formulation, especially hormone concentration and composition^4^, the asexual propagation system suitable for one loquat variety can not be directly copied and extended to other varieties, most importantly, even if asexual plant regeneration is successful, it will take another 7–8 years to grow into adult fruit trees. Therefore, grafting is still one of the most important methods for the expansion of varieties nowadays. Generally, under field conditions, fruit setting can start in the third year after grafting, rootstocks need to grow for approximately 2–3 years before they can be used for grafting, which greatly limits the breeding efficiency of loquat. However, the importance of fast-growing cultivation techniques of loquat rootstocks has not been paid enough attention.

The diameter of main stem is the primary factor to measure the growth and quality of rootstock^5,6^. Besides, it was reported in apple^6,7^ and citrus^8^ that growing indicators such as plant height, crown width, branch number, leaf number per plant, and the leaf area index of Idesia were significantly positively correlated with stem diameter of rootstocks. However, there was also contrary opinion that rootstocks that were too thick might slow the graft unions healing and reduce the survival rate of grafting^8^. In addition, a third view suggested that grafting survival rate and scion growth may be related to the maturity of rootstocks^9^. All of these views indicated that not only the stem diameter, but also the lignification degree of rootstock can affect rootstock quality.

Fertilization plays an important role in promoting the initial growth and improving the quality of rootstock^10,11^, for example, Silva^12^ found that superphosphate promoted the height and stem diameter of plants to the greatest extent after 30 d of transplanting. Ciriello^13^ reported that a there was a trend of increase in plant height and leaf area with addition of nitrogen (N), while the stem diameter and root dry mass data showed a negatively linear way. Tomislav^14^ reported that application of organic fertilizer can increase the content of total nitrogen (TN) and available phosphorus (AP) in the plowing layer soil and promote the root development of grape. All of these results suggested that optimum form, content and proportion of nutrient elements might play a crucial role in regulating soil state and improve plant growth. However, little information is available on the effects of different formulas of fertilizer on the initial growth of rootstock seedlings, reports on the relationships between fertilizer applications and the stem lignification process and the root morphogenesis are also rare. The objective of this study is to find out the optimal fertilization formula which can maintain the most vigorous growth as well as the most suitable lignification process, to provide a guidance for the rapid-growing cultivation technology of loquat rootstocks.

## Results

### Effects of different fertilizer formulas on the height of loquat seedlings

**Figure 1:**
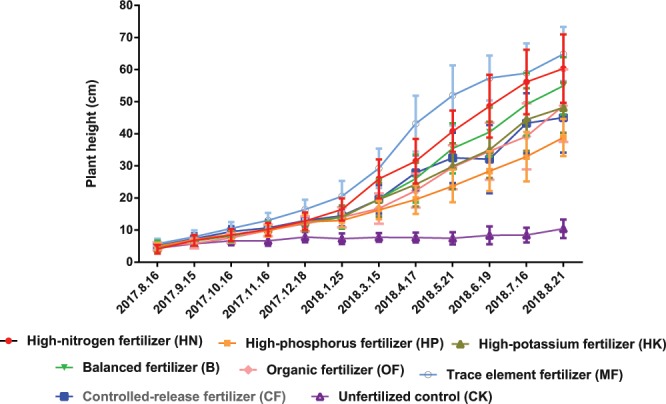
Comparison of loquat rootstock seedling growth under different fertilizer treatments.

As shown in Fig. 1 and Table supplement 1, increase the proportion of N in fertilizer properly or adding trace elements can promote the shoot growth of loquat rootstock. At the end of this experiment, the plant height of the HN-treated loquat rootstocks was 60.33 cm, which was almost 14 times higher than its initial value, while the plant height in the MF treatment was 64.86 cm and 11.6 times higher than the initial values. From January to March, the plant height of the HN-treated seedlings showed the highest increase (57.9%) during this period, and the second was shown by the MF-treated seedlings (42.5%). The minimum was 29.6% under the HP treatment, while the increase in CK was only 5.6%.

### Effects of different fertilizer formulas on loquat stem thickening

**Figure 2:**
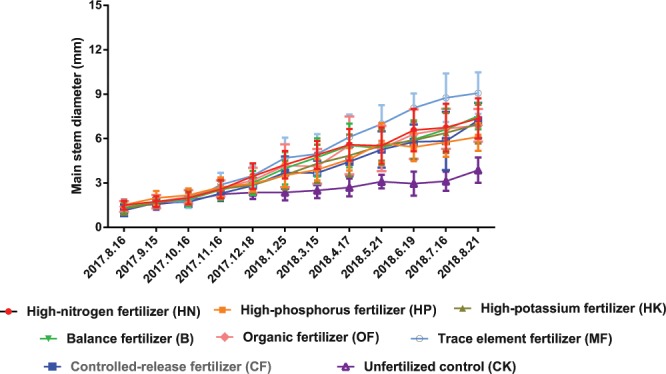
Comparison of loquat stem development under different fertilizer treatments.

It is shown in Fig. 2 and Table supplement 2 that significant differences began to occur between fertilizer treatments and the CK since the third month after transplantation. At the end of the experiment, the stem diameter of the rootstock seedlings treated with MF and HN reached 2.35 and 2.03 times that of the CK, respectively. MF-treated seedlings showed the largest stem diameter (9.07 cm), which is 4.8 times the initial value. The HN-treated seedlings ranked second (7.36 cm), which was 3.3 times the initial value. The fastest growing period of the stem occurred from December to January, and the largest increase was 34.5% under the MF treatment. By contrast, that in the CK was only 0.4%, which means that the stem thickens fastest in winter and that appropriate addition of trace elements may be beneficial to promoting main stem thickening.

### Effects of different fertilizer formulas on loquat leaf growth and development

As shown in Table 1, B-treated seedlings had the largest leaf area, HP-treated seedlings had the smallest leaf area and the least number of leaves, the SPAD value of leaves was the highest under CF treatment and the lowest under HK treatment. These results suggested that slow-release fertilizer is beneficial to N accumulation and chlorophyll synthesis in loquat leaves, the balanced fertilizer showed greatest promotion on leaf growth.

**Table 1:** Comparison of leaf growth and development under different fertilizer treatments.

Treatment	Leaf shape		Leaf area (cm^2^)	Number of leaves per plant	SPAD value of the leaves
	Longitudinal diameter (cm)	Transverse diameter (cm)			
HN	24.33 ± 1.47 c	8.89 ± 0.71 a	132.26 ± 14.18 b	22.38 ± 0.72 a	53.91 ± 0.90 ab
HP	21.11 ± 1.42 d	7.12 ± 0.62 b	103.27 ± 5.50 c	15.62 ± 0.45 d	47.36 ± 1.77 b
HK	25.77 ± 0.35 ab	9.38 ± 0.24 a	148.44 ± 6.62 ab	17.43 ± 0.98 c	34.89 ± 11.33 c
B	27.20 ± 0.56 a	9.20 ± 0.10 a	155.41 ± 3.50 a	18.77 ± 0.61 c	51.18 ± 0.93 ab
OF	25.18 ± 0.44 bc	9.30 ± 0.41 a	142.05 ± 9.36 b	19.47 ± 1.24 bc	50.10 ± 2.58 ab
MF	25.08 ± 0.99 bc	9.55 ± 0.08 a	146.9 ± 9.17 ab	19.60 ± 0.34 b	50.71 ± 0.81 ab
CF	24.45 ± 1.60 bc	8.97 ± 0.73 a	141.68 ± 14.96 ab	15.57 ± 1.07 d	57.35 ± 2.45 a
CK	9.88 ± 0.60 e	3.90 ± 0.41 c	21.83 ± 1.37 d	7.61 ± 0.58 e	26.17 ± 1.88 d

The data are the means ± SDs. The different letters indicate a significant difference (*P* < 0.05).

### Effects of different fertilizer formulas on root morphological development

**Figure 3:**
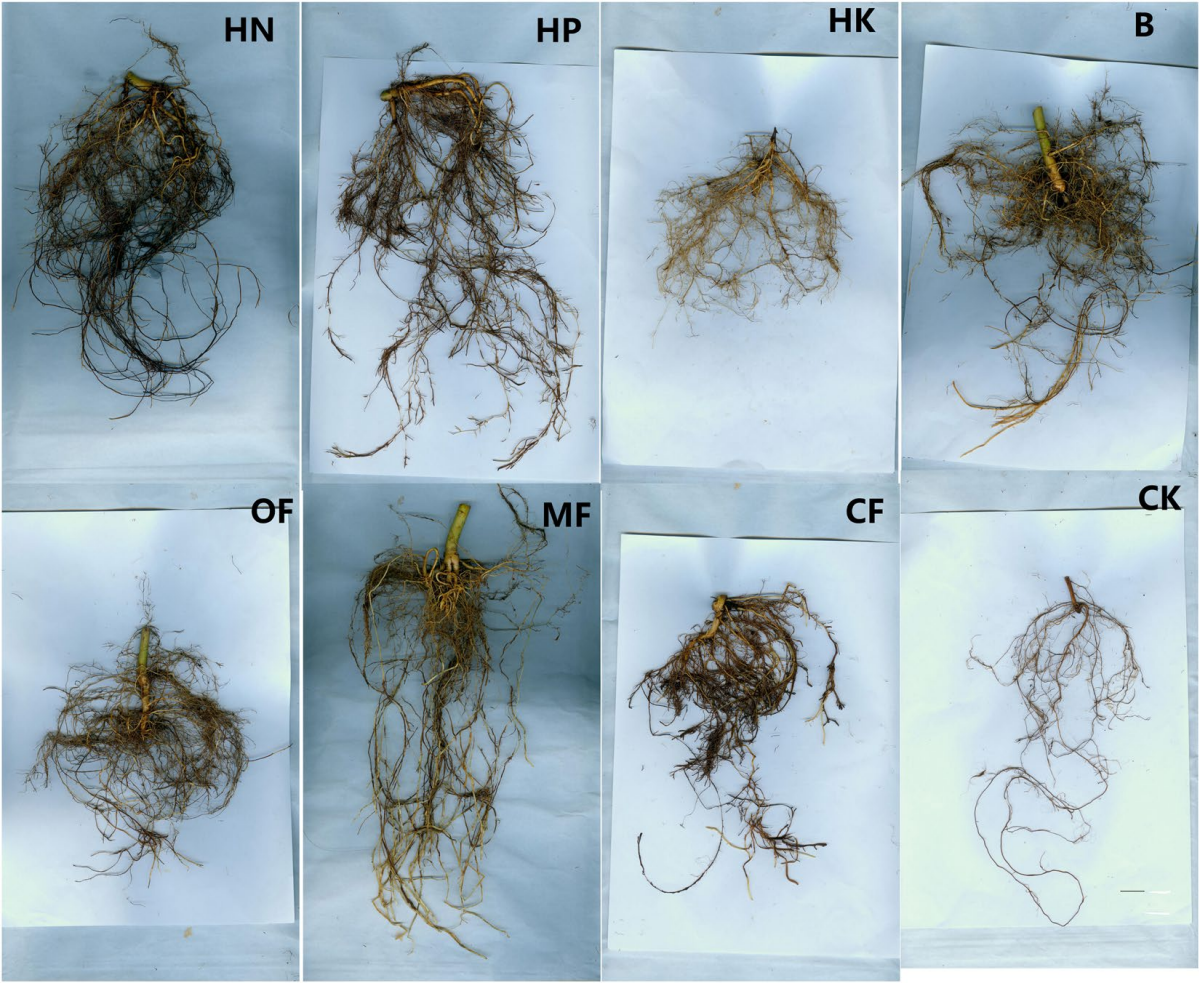
Root morphological differences of rootstock seedlings under different treatments (bar = 1 cm),.

As showed in Fig. 3 and Table 2, the comprehensive level of root development was the highest under the treatment of HN, but the lowest under the treatment of HK. while the average root diameter of the HP-treated seedlings was the smallest, which might mean that there is different ways of N and P promoting root development, N increases root growth quantity and makes root thicker, P promotes a large number of fibrous roots, increase the surface area of roots, and improve the absorption capacity of roots to water and nutrients, too much K will weaken root development.

**Table 2:** The relevant data of root morphology.

Treatment	Total Length (cm)	Total Surface Area (cm^2^)	Average Diameter (mm)	Root Volume (cm^3^)
HN	286.57 ± 12.65 a	20.70 ± 2.34 a	1.88 ± 0.25 a	11.70 ± 0.24 a
HP	300.92 ± 35.24 a	21.04 ± 3.33 a	0.80 ± 0.14 d	13.99 ± 0.05 a
HK	143.10 ± 24.10 d	11.49 ± 1.65 c	0.59 ± 0.11 e	1.46 ± 0.11 g
B	245.12 ± 18.22 b	19.44 ± 5.21 a	0.97 ± 0.21 cd	3.44 ± 0.08 de
OF	302.79 ± 21.14 a	20.24 ± 4.56 a	1.57 ± 0.19 b	10.41 ± 0.14 ab
MF	244.96 ± 14.74 b	17.30 ± 2.41 ab	1.18 ± 0.20 c	5.74 ± 0.22 c
CF	290.58 ± 23.74 a	20.34 ± 5.02 a	0.94 ± 0.24 cd	4.39 ± 0.36 cd
CK	220.46 ± 24.11 bc	16.28 ± 4.85 ab	0.81 ± 0.10 d	2.53 ± 0.42 f

The data are the means ± SDs. The different letters indicate a significant difference (*P* < 0.05).

### Comparison of dry matter accumulation of rootstock seedlings under different treatments

As shown in Table 3, different fertilization formulas could affect biomass accumulation. MF-treated seedlings resulted in the highest stem fresh weight, HN-treated seedlings resulted in the highest fresh weight of leaves and roots, the OF treatment led to the highest dry matter accumulation in leaves, the HP treatment led to the highest dry matter accumulation in the roots.

**Table 3:** Comparison of dry matter accumulation of different parts of loquat seedlings under different fertilizer treatments.

Treatment	Fresh weight of aboveground parts		Fresh weight of roots (g)	The dry/fresh weight ratio		
	stems (g)	leaves (g)		stems	leaves	roots
HN	31.05 ± 3.55 b	57.83 ± 4.51 a	14.23 ± 0.20 a	0.35 ± 0.02 b	0.40 ± 0.01 de	0.33 ± 0.02 ab
HP	13.20 ± 0.77 d	26.38 ± 0.88 d	5.95 ± 0.37 e	0.36 ± 0.02 b	0.42 ± 0.02 cd	0.43 ± 0.06 a
HK	21.94 ± 2.18 c	36.10 ± 4.74 cd	7.32 ± 0.84 de	0.31 ± 0.03 bc	0.38 ± 0.02 e	0.36 ± 0.01 ab
B	25.88 ± 1.83 bc	47.61 ± 1.73 ab	8.61 ± 1.45 bc	0.32 ± 0.01 bc	0.38 ± 0.01 e	0.30 ± 0.01 b
OF	22.22 ± 1.01 c	42.18 ± 2.65 bc	9.20 ± 0.59 cd	0.26 ± 0.01 c	0.60 ± 0.02 a	0.31 ± 0.06 b
MF	38.03 ± 1.51 a	47.70 ± 2.33 ab	11.46 ± 0.68 b	0.35 ± 0.05 b	0.45 ± 0.02 bc	0.34 ± 0.02 b
CF	23.41 ± 2.41 c	37.06 ± 4.88 d	8.08 ± 0.48 d	0.30 ± 0.02 bc	0.38 ± 0.02 e	0.37 ± 0.05 ab
CK	1.37 ± 0.29 e	1.80 ± 0.21 e	3.31 ± 0.24 f	0.47 ± 0.07 a	0.47 ± 0.01 b	0.38 ± 0.08 ab

The data are the means ± SDs. The different letters indicate a significant difference (*P* < 0.05).

### Effects of different fertilizer formulas on the spatial distribution of nutrients in seedlings

The spatial distribution of N, P and K in dried loquat root, stem and leaf samples was also determined. The data showed that the accumulation of N and P in the roots, stems and leaves of loquat rootstock seedlings was the highest under the CF treatment, and the K accumulation in roots, stems and leaves of the loquat rootstock seedlings was highest under the HK treatment (Table 4), which might indicate that plants have synergistic uptake and utilization mechanisms of N and P but independent pathway of K uptake. However, unexpectedly, the N accumulation in the roots, stems and leaves of plants under the HN treatment was the lowest among all fertilizer treatments, which may be related to the easy leaching loss of water-soluble N.

**Table 4:** The concentration and spatial distribution of NPK in loquat seedlings.

	Treatment	Total nitrogen content (TN, %)	Total phosphorus content (TP, %)	Total potassium content (TK, %)
Leaf	HN	2.42 ± 0.49 c	0.10 ± 0.02 bc	1.27 ± 0.21 b
	HP	3.12 ± 0.18 b	0.13 ± 0.05 b	1.28 ± 0.17 b
	HK	2.97 ± 0.14 b	0.13 ± 0.03 b	1.77 ± 0.06 a
	B	2.88 ± 0.11 b	0.07 ± 0.01 cd	1.37 ± 0.42 ab
	OF	2.87 ± 0.11 b	0.08 ± 0.01 cd	1.71 ± 0.19 ab
	MF	2.98 ± 0.26 b	0.09 ± 0.02 bcd	0.72 ± 0.05 c
	CF	3.58 ± 0.16 a	0.20 ± 0.02 a	1.53 ± 0.17 ab
	CK	1.76 ± 0.18 d	0.05 ± 0.01 d	1.27 ± 0.42 b
Stem	HN	2.29 ± 0.47 c	0.32 ± 0.08 abc	1.00 ± 0.04 d
	HP	2.16 ± 0.13 c	0.30 ± 0.13 bc	2.28 ± 0.65 ab
	HK	2.49 ± 0.27 c	0.16 ± 0.07 c	2.37 ± 0.38 a
	B	2.29 ± 0.11 c	0.37 ± 0.12 ab	1.71 ± 0.14 bc
	OF	2.38 ± 0.19 c	0.43 ± 0.12 ab	2.20 ± 0.44 ab
	MF	1.88 ± 0.16 c	0.32 ± 0.07 abc	1.67 ± 0.18 bc
	CF	4.45 ± 0.44 a	0.53 ± 0.14 a	1.73 ± 0.21 bc
	CK	3.30 ± 0.73 b	0.49 ± 0.15 ab	1.35 ± 0.18 cd
Root	HN	1.33 ± 0.45 de	0.14 ± 0.04 b	0.39 ± 0.07 d
	HP	2.52 ± 0.28 b	0.38 ± 0.07 a	0.83 ± 0.11 ab
	HK	2.32 ± 0.15 bc	0.28 ± 0.06 ab	0.97 ± 0.17 a
	B	1.91 ± 0.20 bcd	0.39 ± 0.07 a	0.74 ± 0.14 abc
	OF	1.63 ± 0.05 de	0.18 ± 0.09 b	0.66 ± 0.20 bcd
	MF	1.77 ± 0.08 cd	0.37 ± 0.14 a	0.54 ± 0.09 cd
	CF	3.41 ± 0.74 a	0.42 ± 0.09 a	0.70 ± 0.24 abc
	CK	1.03 ± 0.18 e	0.18 ± 0.04 b	0.65 ± 0.06 bcd

The data are the means ± SDs. The different letters indicate a significant difference (*P* < 0.05).

### Effects of different fertilizer formulas on lignification of stems and roots

**Figure 4:**
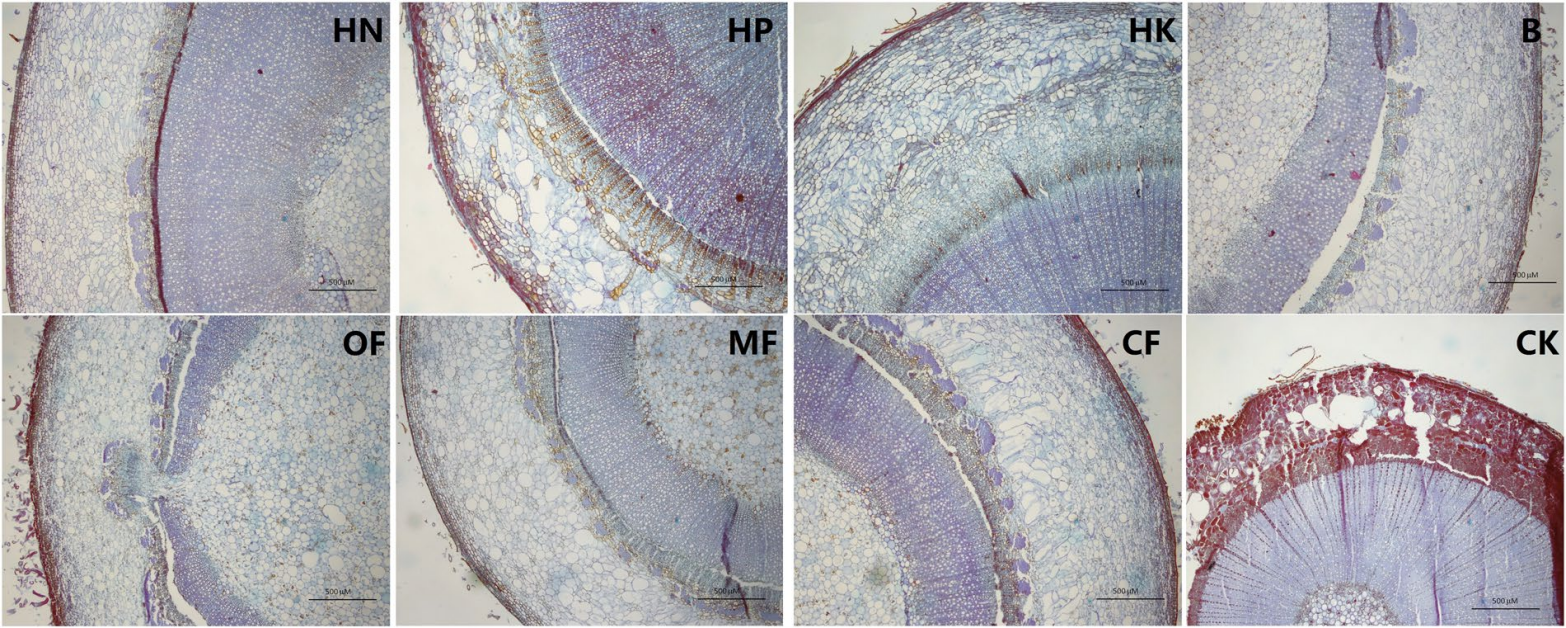
Observations of the cross-sectional anatomical structures of loquat stems under different treatments (bar = 500 μm).

As shown in Fig. 4, saffron staining was obvious in the cambium and periderm under the HN treatment (Fig. 4HN) and in cambium and the secondary xylem under the HP treatment (Fig. 4HP), the stem tissue under the MF B and HK treatments showed relatively slighter lignification in the cambium and periderm compared to that under the other fertilizer treatments, while the lignification in the CK was the most severe, as whole cambium and secondary phloem were dyed red.

**Figure 5:**
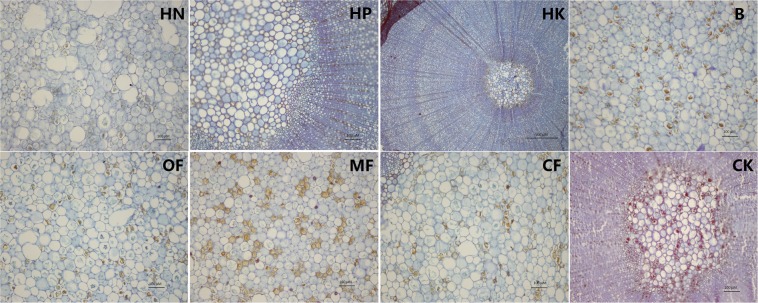
Observations of the cross-sectional anatomical structures of stem pith under different treatments (bar = 100 μm; HK: bar = 500 μm).

Furthermore, it was found that the saffron staining of stem pith cells (red punctuate staining) was deeper under the B, MF and CF treatments than under the other fertilizer treatments. The HN, HP and HK treatments resulted in significantly less lignification of the pith cells (Fig. 5). Thus, it was inferred that compared to NPK balanced fertilizer, relatively higher proportion of N, P or K might slow down the lignification process of stem pith cells.

**Figure 6:**
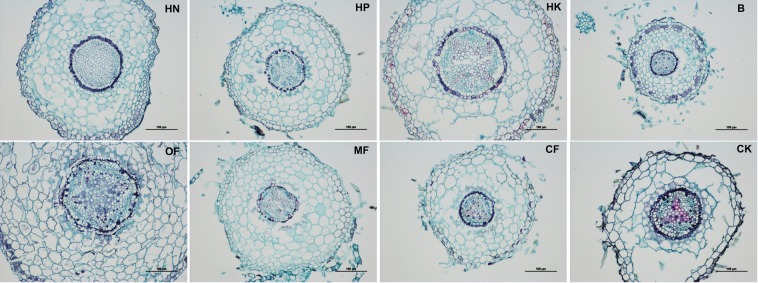
Observations of the cross-sectional anatomical structures of roots under different treatments (bar = 100 μm).

The roots via the paraffin sections was also observed (Fig. 6), the HP treatment resulted in the lowest degree of root lignification while under B treatment, the degree of root lignification was the most serious, especially in the cambium. OF and CF treatment triggerd lignification mainly located in pith tissue.

### Effects of fertilizer formulas on lignin accumulation in the stems and roots

**Figure 7:**
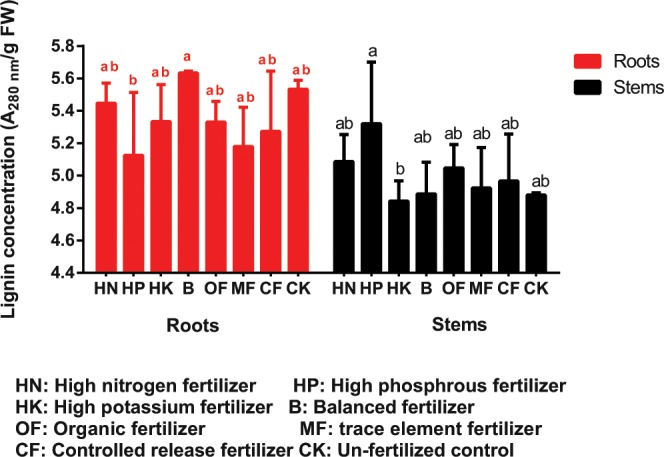
Lignin concentrations in loquat roots and stems under different treatments.

It can be seen from Fig. 7 that HP-treated loquat seedlings presented the highest lignin accumulation in the stems but the lowest in the roots, in contrast, the B treatment led to the highest lignin accumulation in the roots, and the HK treatment resulted in the lowest lignin accumulation in the stem. On the basis of these results and the data in Table 4, it should be noticed that HP, HK and B treatment all led to relatively higher accumulations of N and K in the aboveground part (both leaf and stem) as compared to those in roots, from which we can infer that although lignification of root and stem weakens the rootstock’s ability of nutrient absorption and transportation, it has little effect on nutrient elements with strong mobility, such as N and K, while P accumulated preferentially in root system because of its inconvenient movement in plants.

The data are the means ± SDs. The bars (i.e., the means) with different letters are significantly different within the different parts of plants and different fertilizer treatments (*P* < 0.05).

### Correlation analyses of growth-related indicators of loquat rootstock seedlings

The results of correlation analyses and a principal component analysis showed that plant height (contribution rate of 65.076%), main stem diameter (contribution rate of 19.143%) and leaf SPAD values (contribution rate of 8.577%) best represented the differences in growth and development of loquat rootstock seedlings under the different fertilizer treatments (Table 5). Moreover, it was shown in Table 6 that N and P accumulation in the roots and leaves were significantly correlated, while K accumulation in the roots and stems was significantly correlated. These results may imply that there may be different pathways for N and P uptake and K uptake in loquat, as N and P accumulate preferentially in the leaves, while K accumulates preferentially in the stems.

**Table 5:** The eignvalues, proportions and cumulative contribution rates of principal components.

Principal Component	Indexes	Eigenvalue	Contribution rate (%)	Cumulative contribution rate (%)
1	Plant Height	7.158	65.076	65.076
2	Stem Diameter	2.106	19.143	84.219
	SPAD Value of Leaves	0.943	8.577	92.796
	Leaf Area	0.417	3.795	96.591
	Fresh Weight of Stems	0.258	2.343	98.934
	Fresh Weight of Leaves	0.076	0.692	99.627
	Fresh Weight of Roots	0.041	0.373	100.000
	Average Root Diameter	0.000	0.000	100.000
	Total Surface Area	0.000	0.000	100.000
	Root Volume	0.000	0.000	100.000
	Total Root Length	0.000	0.000	100.000

**Table 6:** Correlation matrix of the spatial distribution of N, P, and K and the lignification of loquat rootstock seedlings under different fertilizer treatments.

		Root N	Stem N	Leaf N	Root P	Stem P	Leaf P	Root K	Stem K	Leaf K	Lignification of Roots	Lignification of Stems
Root N	Pearson Correlation Coefficient	1										
Stem N	Pearson Correlation Coefficient	0.361	1									
Leaf N	Pearson Correlation Coefficient	0.713**	0.196	1								
Root P	Pearson Correlation Coefficient	0.646**	0.081	0.593**	1							
Stem P	Pearson Correlation Coefficient	−0.208	0.014	−0.134	0.113	1						
Leaf P	Pearson Correlation Coefficient	0.761**	0.457*	0.727**	0.325	−0.337	1					
Root K	Pearson Correlation Coefficient	0.460*	0.111	0.227	0.268	−0.124	0.168	1				
Stem K	Pearson Correlation Coefficient	0.358	−0.100	0.449*	0.241	0.005	0.255	0.592**	1			
Leaf K	Pearson Correlation Coefficient	0.141	0.282	0.145	−0.216	−0.154	0.235	0.352	0.317	1		
Lignification−Roots	Pearson Correlation Coefficient	−0.377	0.203	−0.344	−0.185	0.067	−0.401	0.027	−0.283	0.042	1	
Lignification-Stems	Pearson Correlation Coefficient	0.037	−0.150	0.035	0.134	−0.182	0.070	0.157	0.340	0.182	−0.096	1

*and **indicate a significant correlation at *P* < 0.05 and an extremely significant correlation at *P* < 0.01, respectively, between different plant nutrient indexes.

## Discussion

Plant height, main stem diameter and degree of lignification are traditionally considered important indexes for measuring rootstock quality, although many studies have shown that plant growth, mineral nutrient utilization and root morphology construction are mainly affected by genotype^15–17^, the role of exogenous nutrient supply should not be neglected. For example, He^18^ reported that increasing K level in the soil resulted in overall uptake of N, K, Cu and Zn and reduced P, Ca, Mg, Fe and Mn concentrations in cacao plants. Cai^19^ reported that N addition significantly increased *Arabidopsis thaliana* stem diameter, cortical thickness, flower ring radius, midrib thickness, and leaf and stem vascular size, while phosphorus addition significantly increased the stem xylem thickness. Reig^20^ reported that root development in adult loquat trees is controlled by the fruit load, mediated by competition for carbohydrates. These findings indicated that different plants have different optimal ranges of nutrient demands, different concentrations, different ratios, and even different nutrient availability can have quite different effects on plant growth and development.

The role of N promoting fast plant growth is known since decades, however, most of the previous researches were focus on the grafted seedling, in other words, the interaction between rootstocks and scions^21–25^, while our research mainly focused on rootstock itself. It was found in the present study that the shoot height and stem diameter of rootstocks under HN treatment were slightly lower than MF treatment (Figs. 1–2). Meanwhile, HN treatment resulted relatively smaller leaf areas, lower SPAD values and contents of NPK as compared to CF treatment (Tables 1, 3–4), which indicated that the excessive N supply could not continuously promote the vegetative growth, we speculate that excessive N may reduce N utilization efficiency of plants^13,21^. However, previous studies on pear^22^ and grape^25^ showed opposite results that increasing N can continuously and significantly increase leaf area, but it leads to relatively lower NPK accumulation in leaves due to the dilution effect.

P is generally considered can significantly promote root development, in the present study however, it was found that the effect of P on root development is significantly affected by N form. HN treatment had the greatest proportion of urea-N, while the HP treatment had only ammonium-N (Table 7), rootstocks under HP treatment showed the longest root length, the largest root surface area, the greatest root dry mass and root volume, the largest amount of fibrous roots, but relatively the lowest root fresh weight. HN treatment, by contrast, promoted not only fully developed roots, but also highest root fresh weight (Fig. 3, Tables 2–3). These results suggested that P together with ammonium-N can significantly promote the development of fibrous roots, but excessive ammonium-N may weaken the root system and inhibit the shoot growth of loquat rootstock. it was reported in peach^24^ that the increase of ammonium-N absorption is beneficial to the dry matter accumulation of roots, but too much ammonium-N is not conducive to the thickening of stem, this is consistent with our results, while in grape^25^, both ammonium-N and nitrate-N showed less promoting effects on increasing root/shoot ratio than that of amino acid-N. These findings suggested that different plants might have different preferences for nutrient uptake and different ways of transforming and using mineral nutrients.

**Table 7:** Contents and ratios of nutrient elements in the fertilizer treatments.

Treatment	NPK analysis	Total Nitrogen (%)	Ammonium- nitrogen (%)	Nitrate-nitrogen (%)	Urea nitrogen (%)	Water-soluble anhydride (%)	Water- soluble potassium (%)	Water-soluble magnesium (Mg, %)	trace elements					
									Boron (B, %)	Copper (Cu, %)	Manganese (Mn, %)	Molybdenum (Mo, %)	Zinc (Zn, %)	Iron (Fe, %)
HN	30-10-10	20%	5%	5%	24.6%	10%	10%	0.05%						
HP	9-45-15	9%	9%			45%	15%	0.05%						
HK	15-10-30	15%	2%	3%	10%	10%	30%	0.05%						
B	15-15-15	15%	5%	5%		15%	15%	0.05%						
OF		1.63%				1.54%	0.85%							
MF	15-15-15	15%	5%	5%		15%	15%	0.05%	0.0125%	0.05%	0.025%	0.005%	0.0025%	0.05%
CF	15-15-15	15%	5%	5%		15%	15%	0.05%	0.0125%	0.05%	0.025%	0.005%	0.0025%	0.05%
CK	Unfertilized control													

The effect of trace element fertilizers on stem thickening was especially obvious, even exceeding that of the HN treatment (Table 2). It showed in Fig. 4 that the lignification degree of the stem cambium was the highest under the HN treatment, but the lowest under the MF treatment, it could be inferred that excessive N accelerates the stem cambium lignification process, while microelement fertilizer can effectively delay it, thereby increasing the meristematic ability of cambial cells and promoting the thickening of stem.

What is more, our results showed that application of organic fertilizer seemed to be effective in improving dry matter accumulation in leaves (Table 3). Previous studies also provided similar results, for example, Milošević^26^ reported that NPK alone increased leaf P of apple, whereas NPK mixed with cattle manure increased leaf P, K and Mg contents. Nazir^27^ reported that inorganic NP fertilizer mixed with poultry manure improved not only fruit soluble solids and dry matter content, but also plant height and plant spread of strawberry more significantly than inorganic NP fertilizer alone.

In addition to stem thickness, the degree of rootstock lignification is also an important factor affecting rootstock quality. For example, Priyanka^9^ suggested that 7 months old rootstocks of jackfruit recorded the maximum graft success (72.39%) and 5 months old rootstock recorded the least (23.60%), while 4 months old rootstock showed largest number of buds and branches, length and girth of shoot. It indicate that the age and lignification degree of rootstock are not necessarily positively related to the quality of rootstock and the survival rate of grafting. In the present study, the annual rootstocks was insufficient for grafting, thus only the effect of fertilization on the stem lignification process was observed. Figures 4–7 showed that HP treatment triggered highest degree of lignification in stems and greatest lignin accumulation (including xylem ray area and cambium) as well as the lowest degree of lignification and lignin accumulation in roots, which corresponds to the smallest root diameter under HP treatment shown in Table 2. In contrast, B treatment triggered highest degree of lignification and greatest lignin accumulation in roots (Figs. 6–7), as well as the largest leaf area (Table 1). To some extent, the nutrients accumulated by leaf assimilation make up for the deficiency in nutrient absorption capability of the roots, thus ensuring that the overall nutrients of the rootstocks under the B treatment can still be maintained at a moderate level. HK treatment resulted in the lowest lignin concentration is stem (Fig. 7) as well as the weakest root development (Table 2), which indicated that too much K may lead to a decrease in the nutrient uptake capability of the roots, thus reducing the overall plant growth.

In conclusion, the most significant changes of loquat rootstock seedlings under fertilization were plant height and stem diameter, the absorption of N and P by seedlings shows a positive synergistic effect. Increasing the proportion of N in fertilizer can markedly promote the rapid growth and new shoots sprouting of rootstocks. Furthermore, urea-N showed greatest promotive effect on vegetative growth and main stem lignification, while excessive ammonium-N may inhibit shoot growth. Increasing the proportion of phosphorus can significantly promote the occurrence of fibrous roots and improve the water and nutrient absorption capability of the roots. Increasing the K nutrition can reduce the degree of lignification of the stem cambium and delay the stem lignification process; however, excessive K is not conducive to root development and perhaps could inhibit vegetative growth. Trace element fertilizer can delay the lignification process of the stem cambium, which is beneficial to improving loquat rootstock thickness. On the other hand, long-lasting fertilizers such as CF and OF can promote the accumulation of both mineral nutrients and dry matter in plants. These conclusions can provide a guidance to improve the rapid breeding technology of loquat rootstocks.

## Material and Methods

### Tested seedling preparation

We collected loquat seeds from mature fruits beginning at the end of May in 2017. After 10–15 d of artificial germination, thousands of the germinated seeds were transplanted into the plug plate and cultured for 8 weeks, then the seedlings with the similar growth potential were selected as the experimental materials and transplanted into the 1.5 L flowerpots (upper diameter of 16 cm, bottom diameter of 11 cm, height of 14 cm, one seedling of each pot) in which peat (pH = 6.5) served as the cultivation medium. The experimental design was a randomized block with three replications, and each replicate comprised 30 selected seedlings.

### Treatments and experimental design

The whole experiment was carried out in a greenhouse located in Feng Xian district, Shanghai Academy of Agricultural Sciences in Shanghai, China. In order to simulate the conventional field fertilization as much as possible, we selected commercial fertilizers in this experiment and determined the nutrient element content and proportion of each fertilization formula, finally eight fertilizer treatments were established (Table 7), including five water-soluble fertilizer formulas (HN (high-nitrogen fertilizer), HP (high-phosphorus fertilizer), HK (high-potassium fertilizer), B (balanced fertilizer) and MF (trace element fertilizer, B + trace elements)), as well as an organic fertilizer (OF) and a granular controlled-release fertilizer (CF). An unfertilized control (CK) was also included.

### Fertilization frequency

The loquat seedlings were treated with 1 g of water-soluble fertilizer (HN, HP, HK, B, MF) per pot once a week from August to October and with 2 g per pot once every two weeks from November to April; the amount was further increased to 5 g per pot once every two weeks from May to August. OF and CF were directly mixed into the cultivation substrate once every 3 months (25 g per pot) separately, while the CK treatment received water only.

### Monitoring index

The plant height, main stem diameter, leaf number, and leaf SPAD values were measured once a month, and the leaf area, dry and fresh weight of the above/underground parts, root morphological characteristics, lignin content of the roots and stems and nutrient element spatial distribution in the plants were analyzed at the end of the experiment. Furthermore, we also observed the anatomy of the loquat stems by paraffin sections.

### Determination methods

The plant height and longitudinal and transverse diameters of the leaf blades of the loquat seedlings were measured by a ruler, the stem diameter was measured by a Vernier caliper, the leaf SPAD values were measured with a chlorophyll meter (SPAD-502 Plus, Minolta Camera Co., Ltd., Japan), and the leaf area was measured with a portable area meter (Li-3000C, LI-COR, USA).

The root morphology of the seedlings was scan, image and analyzed via the GXY-A root analyzer.

The content of total nitrogen (TN) was determined by the Kjeldahl method, total phosphorus (TP) content was determined by the colorimetric method with ammonium molybdate, and the total potassium (TK) content was determined by flame photometry^28^. The lignin concentration was determined according to the method of Hu^29^.

### Statistical analysis

Each experiment involved a completely randomized design with three independent replicates for each treatment. Statistical analysis (Student’s t-test and ANOVAs) and graphing were performed using Prism 4 (GraphPad, Lo Jolla, CA), principal component analysis (PCA) and correlation analyses were carried out via SPSS 18.0 (SPSS Inc., Chicago, IL). Differences were considered statistically significant when *P* < 0.05.

## Supplementary information

Supporting Information.
