# Supplementary material for: Effects of different fertilizer formulas on the growth of loquat rootstocks and stem lignification
Source: Sci Rep. 2020 Jan 23;10:1033. doi: 10.1038/s41598-019-57270-5 (PMC6978351; doi:10.1038/s41598-019-57270-5)
Supplement: Supplementary file 1 — Supporting Information. [file 41598_2019_57270_MOESM1_ESM.pdf]

Title：Effects of different fertilizer formulas on the growth of loquat rootstocks and stem lignification

(SREP-19-29151B)

Fangjie Xu[1] Changbin Chu[2], Zhihong Xu[3*]

[1]Forestry and Pomology Research Institute/Shanghai Key Lab of Protected Horticultural Technology, Shanghai Academy of Agricultural Sciences, No. 1000 Jinqi Road, Fengxian District, Shanghai 201403, P. R. China

[2]Eco-Environmental Protection Institute, Shanghai Academy of Agricultural Sciences, No. 1000 Jinqi Road, Fengxian District, Shanghai 201403, P. R. China

[3]College of Agricultural & Food Science, Zhejiang A & F University, No. 88 North Huancheng Road, Li'an District, Zhejiang, 311300, P. R. China

* Corresponding author: E-mail: zhhxu@zafu.edu.cn

Table supplement 1 Comparison of loquat rootstock seedling growth under different fertilizer treatments

Treatment	2017.8.16	2017.9.15	2017.10.16	2017.11.16	2017.12.18	2018.1.25	2018.3.15	2018.4.17	2018.5.21	2018.6.19	2018.7.16	2018.8.21
HN	4.03±1.35d	6.77±1.61b	8.40±1.89bc	10.27±2.00b	12.80±2.68b	16.40±3.44b	25.93±6.08b	31.47±6.97b	40.79±6.45b	48.63±9.80b	56.14±10.03b	60.33±10.62b
HP	4.93±1.14bc	7.03±1.47b	8.67±1.48bc	9.76±1.84b	12.53±2.15b	12.97±2.11c	16.23±2.87d	19.50±5.24f	23.72±5.05f	28.37±6.21f	32.88±7.16f	38.86±5.79f
HK	4.30±1.15cd	6.83±1.42b	8.53±1.23bc	10.21±1.95b	12.37±2.74b	14.21±3.36c	19.50±5.24c	24.21±6.82de	29.88±7.16de	35.00±8.74d	44.43±9.78d	48.18±7.84d
B	5.07±1.68ab	6.60±1.54bc	8.10±1.93bc	10.38±2.19b	12.28±2.59b	13.93±3.52c	19.68±6.13c	26.04±7.48cd	35.46±7.82c	40.54±7.60e	49.16±9.78c	54.96±8.93c
OF	5.00±1.23bc	5.80±1.52c	7.57±1.61cd	9.90±1.84b	11.97±2.32b	13.97±3.15c	16.69±4.72d	22.30±5.30ef	29.58±6.84e	34.58±8.89de	39.18±10.28e	48.76±11.32d
MF	5.73±1.53a	7.90±2.01a	10.50±1.96a	13.04±2.28a	16.43±3.04a	20.54±4.76a	29.25±6.17a	43.17±8.71a	51.96±9.36a	57.38±6.98a	58.89±9.26a	64.86±8.44a
CF	5.13±1.59ab	7.27±1.68ab	9.56±1.63ab	10.67±2.40b	12.96±2.34b	14.43±3.20c	19.59±4.45c	27.85±5.45bc	32.50±7.86cd	32.10±10.60e	43.28±9.35d	45.07±10.92e
CK	4.37±1.22bcd	5.73±1.36c	6.63±1.37d	6.63±1.54c	7.80±1.58c	7.30±1.66d	7.72±1.35d	7.67±1.54g	7.43±1.89g	8.37±2.77g	8.43±2.28g	10.40±2.86g

*The data in Table supplement 1 are supplemental to the data in Fig. 1. The data are the means±SDs. The different letters indicate a significant difference (*P*<0.05).

Table supplement 2 Comparison of loquat stem development under different fertilizer treatments

Treatment	2017.8.16	2017.9.15	2017.10.16	2017.11.16	2017.12.18	2018.1.25	2018.3.15	2018.4.17	2018.5.21	2018.6.19	2018.7.16	2018.8.21
HN	1.50±0.29ab	1.74±0.37b	1.97±0.42bcd	2.57±0.63bc	3.45±0.87a	4.23±0.93b	4.90±0.95a	5.58±1.07ab	5.51±1.24b	6.57±1.43b	6.74±1.61b	7.36±1.36bc
HP	1.52±0.32a	1.98±0.48a	2.17±0.46b	2.72±0.65ab	2.95±0.52cd	3.51±0.79c	3.89±0.71bc	4.60±0.75c	5.71±1.33b	5.42±0.95c	5.75±0.97c	6.11±0.93b
HK	1.26±0.39cd	1.64±0.31cd	2.01±0.47bc	2.59±0.55ab	2.85±0.52d	3.53±0.97c	4.30±1.29ab	4.85±1.42bc	5.52±1.02b	5.88±1.25bc	6.38±1.35b	6.98±1.31bc
B	1.32±0.37bc	1.72±0.37bc	1.89±0.46bcd	2.53±0.69ab	3.01±0.80c	3.99±1.10bc	4.73±1.29a	5.54±1.47ab	5.40±1.19b	5.93±1.30bc	6.61±1.41b	7.51±0.89b
OF	1.41±0.36abc	1.73±0.46b	2.05±0.46b	2.63±0.56ab	3.18±0.84b	4.16±1.44b	4.12±1.17bc	5.53±1.95b	5.33±1.53b	6.30±1.67bc	6.66±1.36b	6.90±1.10c
MF	1.34±0.37abc	1.71±0.31bc	1.74±0.43cd	2.87±0.81a	3.48±0.70a	4.69±1.37a	4.96±1.33a	6.10±1.52a	6.98±1.28a	8.06±0.98a	8.76±1.64a	9.07±1.41a
CF	1.15±0.40d	1.66±0.29bcd	1.72±0.34d	2.29±0.32cd	2.82±0.52d	3.70±1.09c	3.68±0.84c	4.44±0.99c	5.26±1.23b	5.78±1.17bc	5.83±1.97c	7.22±1.19bc
CK	1.40±0.47bc	1.56±0.37d	1.78±0.38bcd	2.24±0.46d	2.35±0.43e	2.36±0.53d	2.50±0.52d	2.69±0.60d	3.09±0.45c	2.95±0.81d	3.12±0.65d	3.87±0.86e

*The data in Table supplement 2 are supplemental to the data in Fig. 1. The data are the means±SDs. The different letters indicate a significant difference ($P<0.05$).

Table supplement 3 Lignin concentrations in loquat roots and stems under different treatments

Treatment	Roots	Stems
HN	5.45±0.12 ab	5.09±0.17 ab
HP	5.13±0.39 b	5.32±0.38 a
HK	5.33±0.23 ab	4.84±0.12 b
B	5.63±0.01 a	4.89±0.20 ab
OF	5.33±0.11 ab	5.05±0.14 ab
MF	5.18±0.24 ab	4.92±0.25 ab
CF	5.27±0.37 ab	4.97±0.29 ab
CK	5.53±0.06 ab	4.88±0.01 ab

*The data in Table supplement 3 are supplemental to the data in Fig. 7. The data are the means±SDs. The different letters indicate a significant difference (P<0.05).
